# Identification of Classes of Functioning Trajectories and Their Predictors in Individuals With Spinal Cord Injury Attending Initial Rehabilitation in Switzerland

**DOI:** 10.1016/j.arrct.2021.100121

**Published:** 2021-03-15

**Authors:** Jsabel Hodel, Cristina Ehrmann, Anke Scheel-Sailer, Gerold Stucki, Jerome E. Bickenbach, Birgit Prodinger

**Affiliations:** a Swiss Paraplegic Research, Nottwil, Switzerland; b Department of Health Sciences and Medicine, University of Lucerne, Lucerne, Switzerland; c Swiss Paraplegic Centre, Nottwil, Switzerland; d Center for Rehabilitation in Global Health Systems, University of Lucerne, Lucerne, Switzerland; e Faculty of Applied Health and Social Sciences, Technical University of Applied Sciences Rosenheim, Rosenheim, Germany

**Keywords:** Latent class analysis, Logistic models, Longitudinal studies, Observational study, Rehabilitation, Spinal cord injuries, ADL, activities of daily living, AIC, Akaike information criterion, AIS, American Spinal Injury Association Impairment Scale, BIC, Bayesian information criterion, LPMM, latent process mixed model, SCI, spinal cord injury, SCIM III, Spinal Cord Independence Measure version III, SSABIC, sample-size adjusted Bayesian information criterion, SwiSCI, Swiss Spinal Cord Injury Cohort Study

## Abstract

**Objectives:**

To identify classes of functioning trajectories in individuals with spinal cord injury (SCI) undergoing initial rehabilitation after injury and to examine potential predictors of class membership to inform clinical planning of the rehabilitation process.

**Design:**

Longitudinal analysis of the individual's rehabilitation stay using data from the Inception Cohort of the Swiss Spinal Cord Injury Cohort Study (SwiSCI).

**Setting:**

Initial rehabilitation in specialized centers in Switzerland.

**Participants:**

Individuals with newly acquired SCI (N=748; mean age, 54.66±18.38y) who completed initial rehabilitation between May 2013 and September 2019. The cohort was primarily composed of men (67.51%), persons with paraplegia (56.15%), incomplete injuries (67.51%), and traumatic etiologies (55.48%).

**Interventions:**

Not applicable.

**Main Outcome Measures:**

Functioning was operationalized with the interval-based sum score of the Spinal Cord Independence Measure version III (SCIM III). For each individual, the SCIM III sum score was assessed at up to 4 time points during rehabilitation stay. The corresponding time of assessment was recorded by the difference in days between the SCIM III assessment and admission to the rehabilitation program.

**Results:**

Latent process mixed model analysis revealed 4 classes of functioning trajectories within the present sample. Class-specific predicted mean functioning trajectories describe *stable high functioning* (n=307; 41.04%), *early functioning improvement* (n=39; 5.21%), *moderate functioning improvement* (n=287; 38.37%), and *slow functioning improvement* (n=115; 15.37%), respectively. Out of 12 tested factors, multinomial logistic regression showed that age, injury level, injury severity, and ventilator assistance were robust predictors that could distinguish between identified classes of functioning trajectories in the present sample.

**Conclusions:**

The current study establishes a foundation for future research on the course of functioning of individuals with SCI in initial rehabilitation by identifying classes of functioning trajectories. This supports the development of specifically tailored rehabilitation programs and prediction models, which can be integrated into clinical rehabilitation planning.

Spinal cord injury (SCI) is physical damage to the spinal cord with a resulting loss of autonomic, motor and sensory functions below the level of injury, which adversely affects an individual's ability to perform activities and participate in major areas of life.^1^ A newly acquired SCI and its potentially life-changing consequences require a goal-oriented and interdisciplinary rehabilitation process starting as early as possible after the event to optimize an individual's functioning.^2^ Following the World Health Organization's International Classification of Functioning, Disability and Health,^3^ functioning describes the nature and extent of body functions and individual activities that result from an interaction between a health condition and environmental and personal contextual factors. Therefore, initial rehabilitation after SCI not only involves optimizing an individual's neurologic functions, it also addresses an individual's functioning requirements, including optimizing performance and independence in everyday life and adaptation and modification of the environment to enable full participation in the community.

As a result, monitoring functioning outcomes throughout the rehabilitation process is fundamental for individual goal setting, rehabilitation planning, and management, as well as for quality assurance.^4^ Different instruments have been developed to capture an individual's functioning by means of a summary score. The Spinal Cord Independence Measure version III (SCIM III),^5^ for example, describes an individual's independence in activities of daily living (ADL) in mobility, self-care, respiration, and bladder and bowel management and has demonstrated sensitivity to change.^6^ If assessed longitudinally, such functioning sum scores are understood as an individual functioning trajectory (ie, an individual's course of functioning over time). Depending on their demographics,^7^, ^8^, ^9^ injury characteristics,^10^ the occurrence of complications,^10^ and the availability of rehabilitation services,^11^ people may develop differently during their initial rehabilitation stay and may show various individual functioning sum scores over time.

A nuanced picture of these heterogeneous individual functioning trajectories during the initial rehabilitation stay, including the identification of homogeneous subgroups of functioning trajectories and their predictors, can help to specifically tailor rehabilitation programs to the individual's functioning needs. In the SCI literature, studies have investigated classes of trajectories of musculoskeletal shoulder pain,^12^ body mass index,^13^ employment status,^14^ life satisfaction,^15^ mental health,^16^ depression,^17^ and self-efficacy and depressed mood^18^ during initial rehabilitation and up to 5 years after discharge. As far as we are aware, no study has yet investigated classes of functioning trajectories assessed by a summary score for functioning such as the SCIM III. Therefore, this study aimed to identify classes of functioning trajectories in individuals with SCI undergoing initial rehabilitation in specialized centers in Switzerland and to examine potential predictors of class membership to inform clinical planning in the rehabilitation process.

## Methods

### Study design and participants

This study used data from the Inception Cohort of the prospective Swiss Spinal Cord Injury Cohort Study (SwiSCI).^19^ The SwiSCI Inception Cohort included individuals with newly acquired and diagnosed SCI who were recruited upon entry to an initial rehabilitation program in a specialized SCI rehabilitation center in Switzerland (SCI Center, Balgrist University Hospital, Zurich; Centre for SCI and Severe Head Injury, REHAB Basel, Basel; Clinique Romande de Réadaptation, Sion; Swiss Paraplegic Centre, Nottwil). Further inclusion criteria were minimum age of 16 years and permanent residence in Switzerland. Criteria for exclusion were congenital conditions, palliative context, neurodegenerative disorders, or Guillain-Barrée syndrome leading to SCI. A detailed description of the inclusion and exclusion criteria can be found elsewhere.^19^ The longitudinal design of the SwiSCI Inception Cohort included up to 4 time points of data collection during initial rehabilitation stay (T1, 4wk after SCI diagnosis; T2, 12wk after SCI diagnosis; T3, 24wk after SCI diagnosis; T4, at discharge).^20^ The responsible regional ethics committees approved the SwiSCI and all participants gave written informed consent.

Between May 2013 and September 2019, 1182 eligible individuals completed initial rehabilitation in a collaborating rehabilitation center, 1050 of whom consented to the SwiSCI Inception Cohort. For the purpose of comparability between centers and according to the longitudinal study design, we excluded participants based on the following criteria and in specific order: (1) implausible assessment time points of SCIM III (eg, SCIM measurements for T2 were dated to be assessed before the measurements for T1) or individuals whose first assessment occurred within intensive care after SCI (N=52), and (2) fewer than 2 SCIM III assessments during initial rehabilitation stay (N=250). In total, 748 participants were included in this study.

### Measures

#### Main outcome and time of assessment

The main outcome of this study was functioning, which was operationalized by using the SCIM III sum score.^5^ Previously derived interval-based SCIM III sum scores^21^ based on Rasch analysis were used to accurately assess changes in functioning sum scores over time and to allow for their meaningful comparison. These interval-based sum scores range from 0-100, with larger numbers indicating more independence in performing ADL. In the SwiSCI Inception Cohort, the corresponding time of assessment of SCIM III was recorded in days since SCI diagnosis. Because patients spend different lengths of time in acute or intensive care prior to being admitted to initial rehabilitation, days since diagnosis is not representative for the start of inpatient rehabilitation. The respective assessment time points were recalculated into days since admission to the initial rehabilitation program. In what follows, assessment time points with respect to SCIM III refer to days since admission to initial rehabilitation program.

#### Predictors of class membership

Based on expert opinion and previously published studies on predictors of SCIM outcomes, we identified suitable variables collected in the SwiSCI as potential predictors of class membership (methods of assessments are described elsewhere).^22^^,^^23^ Of these, only variables that showed less than 20% missing observations were included in the analysis.

We included the following variables as potential predictors of class membership: age at SCI diagnosis (in years), sex (female, male), language of correspondence (German, French, Italian, other), insurance type (health, disability, accident, self-pay), ward type (basic, semiprivate, private), etiology (traumatic, nontraumatic), injury level (according to the Neurological Classification of Spinal Cord Injury and the neurologic level of injury: tetraplegia, C1-C8; paraplegia, T1-S5; intact) and severity at T1 (according to the Neurological Classification of Spinal Cord Injury and the American Spinal Injury Association Impairment Scale [AIS]: complete, AIS grade A; incomplete, AIS grades B, C, or D; normal, AIS grade E), existence of comorbidities before SCI (any diagnosis other than SCI with diagnosis date before SCI diagnosis, no/yes), requiring ventilation assistance (no/yes), and cardiovascular (no/yes) and pulmonary (no/yes) conditions and complications at T1 since SCI diagnosis. Variables including associated injuries; partner at time of SCI diagnosis; the presence of pain, anxiety, and depression symptoms; normal defecation; urinary tract infections; or pressure injuries were identified as suitable potential predictors but were not included owing to the number of missing observations.

### Data analysis

#### Classes of functioning trajectories

To identify the number of different classes of functioning trajectories within the present sample of individual interval-based SCIM III sum score trajectories, we used latent process mixed models (LPMMs)^24^, ^25^, ^26^ because these models can handle unstructured assessment time points and individuals with different numbers of assessments.^26^ The analysis included 2 steps: (1) Three LPMMs with different parameterized link functions—linear function and quadratic I-splines functions with 2 or 3 knots at percentiles—were estimated to identify the best-fitting link function able to account for non-normal and bounded longitudinal outcomes.^26^ The models were compared using the Akaike information criterion (AIC) and the best-fitting link function was determined by the model with lowest AIC. (2) Two sets of 6 LPMMs, each with an increasing number of latent classes (1-6), were estimated to identify the number of classes of functioning trajectories. In the first set, the specification of the variability of between-person functioning trajectories was fixed across classes. In the second set, this variability was allowed to be proportionally varying across classes. Both sets incorporated the best-fitting link function from step 1. The models were compared using the Bayesian information criterion (BIC), the sample-size adjusted BIC (SSABIC), and the AIC. Better model fit was indicated by lower values for all 3 indices. In addition, they were evaluated and compared according to their convergence, interpretability, entropy indicator describing the degree of class separation (a higher value indicated better separation between classes and therefore better classification accuracy), and class sample sizes according to the most likely class membership (preference for models with class sample sizes including at least 5% of the study participants).

In both steps, all fitted models corresponded to unconditional models (ie, no covariates were integrated). Supplemental appendix S1 (available online only at http://www.archives-pmr.org/) presents detailed model specifications and R syntax of the final LPMM. Alternative model specifications were tested and are available from the authors on request.

#### Predictors of class membership

According to the standard 3-step method,^27^ the following analysis was conducted to examine potential predictors of class membership: (1) the most likely class membership of each participant was extracted from the best-fitting LPMM; (2) the extracted information was merged with the original data; and (3) a multinomial logistic regression was conducted based on the merged data to examine potential predictors of class membership. Before this step, missing observations in potential predictor variables were imputed using the nonparametric random forest method MissForest^28^ and categorical variables were dichotomized. The robustness of the regression analysis was investigated by means of a sensitivity analysis including complete cases only.

All analyses were performed using the software R for Windows, version 3.6.0.^a^ LPMMs were fitted using the R package lcmm (version 1.8.1),^b^ missing data imputation was performed using the R package missForest (version 1.4),^c^ and multinomial logistic regression was conducted using the R package nnet (version 7.3-12).^d^ The study reporting followed the Guidelines for Reporting on Latent Trajectory Studies Checklist^27^ and the Strengthening the Reporting of Observational Studies in Epidemiology statement.^29^

## Results

### Sample characteristics

Of the 748 participants included in this study, 2 SCIM III assessments were available for 408 individuals, 3 for 186 individuals, and 4 for 154 individuals (reasons for <4 assessments include late admission or consent, a short rehabilitation stay, or missing observations). Sample descriptive information including details about the time of assessment of SCIM III are presented in table 1. Participants had a mean age of 54.66±18.38 years, and the cohort was primarily composed of men (67.51%), persons with paraplegia (56.15%), incomplete injuries (67.51%), and traumatic etiologies (55.48%). The median time between SCI diagnosis and admission to initial rehabilitation was 14 days (first quartile, 9d; third quartile, 24d).

**Table 1:** Characteristics of study participants

Characteristics	SwiSCI Inception Cohort Study (N=1050)	Excluded from present study (N=302)	Included in present study (N=748)	P-value
Female, n (%)	342 (32.57)	99 (32.78)	243 (32.49)	0.93
Age at SCI diagnosis, mean ± SD, y	55.21 ± 18.51	56.57 ± 18.81	54.66 ± 18.38	0.16
Length of stay, mean ± SD, d	137.22 ± 82.56	125.99 ± 91.23	141.75 ± 78.40	<0.001
Interval-based SCIM III sum score at T1,				<0.001
median [Q_1_, Q_3_]	71.36 [55.95, 88.62]	42.63 [16.96, 71.36]	73.31 [58.76, 88.69]	
Missing, n (%)	270 (25.71)	230 (76.16)	40 (5.35)	
Interval-based SCIM III sum score at T2,				<0.001
median [Q_1_, Q_3_]	86.11 [69.33, 91.87]	63.79 [51.39, 76.06]	86.84 [72.35, 91.87]	
Missing, n (%)	659 (62.76)	277 (91.72)	382 (51.07)	
Interval-based SCIM III sum score at T3,				<0.01
median [Q_1_, Q_3_]	85.28 [68.82, 91.17]	75.17 [55.95, 82.44]	87.00 [69.35, 91.81]	
Missing, n (%)	851 (81.05)	281 (93.05)	570 (76.20)	
Interval-based SCIM III sum score at T4,				0.04
median [Q_1_, Q_3_]	91.63 [84.29, 95.47]	90.44 [74.24, 95.47]	91.87 [85.33, 95.47]	
Missing, n (%)	183 (17.43)	173 (57.28)	10 (1.34)	
Assessment time point SCIM III T1 since admission to initial rehabilitation program,				<0.001
median [Q_1_, Q_3_], d	11.00 [1.00, 19.00]	-2.50* [-19.25, 4.00]	12.00 [3.00, 20.00]	
Missing, n (%)	266 (25.33)	226 (74.83)	40 (5.35)	
Assessment time point SCIM III T2 since admission to initial rehabilitation program,				<0.001
median [Q_1_, Q_3_], d	68.00 [56.00, 76.00]	51.00 [28.00, 58.00]	69.00 [57.00, 76.00]	
Missing, n (%)	659 (62.76)	277 (91.72)	382 (51.07)	
Assessment time point SCIM III T3 since admission to initial rehabilitation program,				<0.001
median [Q_1_, Q_3_], d	146.00 [134.00, 159.00]	128.00 [116.00, 143.00]	148.00 [137.00, 160.00]	
Missing, n (%)	851 (81.05)	281 (93.05)	570 (76.20)	
Assessment time point SCIM III T4 since admission to initial rehabilitation program,				0.01
median [Q_1_, Q_3_], d	129.00 [69.00, 186.00]	116.00 [28.00, 191.00]	132.00 [72.25, 185.75]	
Missing, n (%)	181 (17.24)	171 (56.62)	10 (1.34)	
Traumatic etiology, n (%)	596 (56.76)	181 (59.93)	415 (55.48)	0.19
Level of injury at T1, n (%)				<0.001
Tetraplegia	333 (31.71)	96 (31.79)	237 (31.68)	
Paraplegia	530 (50.48)	110 (36.42)	420 (56.15)	
Intact	8 (0.76)	2 (0.66)	6 (0.80)	
Missing	179 (17.05)	94 (31.13)	85 (11.36)	
Severity of injury at T1, n (%)				<0.001
Complete	200 (19.05)	53 (17.55)	147 (19.65)	
Incomplete	658 (62.67)	153 (50.66)	505 (67.51)	
Normal	7 (0.67)	2 (0.66)	5 (0.67)	
Missing	185 (17.62)	94 (31.13)	91 (12.17)	
Associated injuries, n (%)				<0.001
No	302 (28.76)	114 (37.75)	188 (25.13)	
Yes	419 (39.90)	121 (40.07)	298 (39.84)	
Missing	329 (31.33)	67 (22.19)	262 (35.03)	
Comorbidities before SCI diagnosis, n (%)				<0.001
No	147 (14.00)	20 (6.62)	127 (16.98)	
Yes	704 (67.05)	112 (37.09)	592 (79.14)	
Missing	199 (18.95)	170 (56.29)	29 (3.88)	
Language of correspondence, n (%)				0.05
German	797 (75.90)	246 (81.46)	551 (73.66)	
French	208 (19.81)	43 (14.24)	165 (22.06)	
Italian	30 (2.86)	10 (3.31)	20 (2.67)	
Other	7 (0.67)	1 (0.33)	6 (0.80)	
Missing	8 (0.76)	2 (0.66)	6 (0.80)	
Insurance type, n (%)				<0.001
Health	536 (51.05)	84 (27.81)	452 (60.43)	
Disability	7 (0.67)	0 (0.00)	7 (0.94)	
Accident	337 (32.10)	56 (18.54)	281 (37.57)	
Self-pay	1 (0.10)	1 (0.33)	0 (0.00)	
Missing	169 (16.10)	161 (53.31)	8 (1.07)	
Ward type, n (%)				<0.001
Basic	477 (45.43)	73 (24.17)	404 (54.01)	
Semi-private	244 (23.24)	41 (13.58)	203 (27.14)	
Private	125 (11.90)	22 (7.28)	103 (13.77)	
Missing	204 (19.43)	166 (54.97)	38 (5.08)	
Partner at time of SCI diagnosis, n (%)				<0.001
No	132 (12.57)	9 (2.98)	123 (16.44)	
Yes	334 (31.81)	29 (9.60)	305 (40.78)	
Missing	584 (55.62)	264 (87.42)	320 (42.78)	
Cardiovascular conditions and complications at T1 since SCI diagnosis, n (%)				<0.001
No	608 (57.90)	84 (27.81)	524 (70.05)	
Yes	262 (24.95)	41 (13.58)	221 (29.55)	
Missing	180 (17.14)	177 (58.61)	3 (0.40)	
Pulmonary conditions and complications at T1 since SCI diagnosis, n (%)				<0.001
No	583 (55.52)	64 (21.19)	519 (69.39)	
Yes	279 (26.57)	57 (18.87)	222 (29.68)	
Missing	188 (17.90)	181 (59.93)	7 (0.94)	
Ventilation assistance at T1 since SCI diagnosis, n (%)				<0.001
No	762 (72.57)	88 (29.14)	674 (90.11)	
Yes	98 (9.33)	30 (9.93)	68 (9.09)	
Missing	190 (18.10)	184 (60.93)	6 (0.80)	
Normal defecation at T1 since SCI diagnosis, n (%)				<0.001
No	317 (30.19)	24 (7.95)	293 (39.17)	
Yes	171 (16.29)	12 (3.97)	159 (21.26)	
Missing	562 (53.52)	266 (88.08)	296 (39.57)	
Urinary tract infection at T1 since SCI diagnosis, n (%)				<0.001
No	368 (35.05)	34 (11.26)	334 (44.65)	
Yes	148 (14.10)	3 (0.99)	145 (19.39)	
Missing	534 (50.86)	265 (87.75)	269 (35.96)	
Pressure injury at T1 since SCI diagnosis, n (%)				<0.001
No	419 (39.90)	27 (8.94)	392 (52.41)	
Yes	104 (9.90)	11 (3.64)	93 (12.43)	
Missing	527 (50.19)	264 (87.42)	263 (35.16)	
Pain at T1 in the past week, n (%)				<0.001
No	94 (8.95)	4 (1.32)	90 (12.03)	
Yes	277 (26.38)	17 (5.63)	260 (34.76)	
Missing	679 (64.67)	281 (93.05)	398 (53.21)	

NOTE. *P* values for distribution comparisons were calculated using the Mann-Whitney *U* test for continuous variables and Pearson chi-square test for categorical variables (both without continuity correction).

Abbreviations: Q_1_, 1st quartile; Q_3_, 3rd quartile; T1-T4, time point of assessment within the SwiSCI Inception Cohort Study.

⁎ For some individuals, the first SCIM III assessment occurred in intensive care after SCI and before admission to the initial rehabilitation program.

### Classes of functioning trajectories

**Fig 1:**
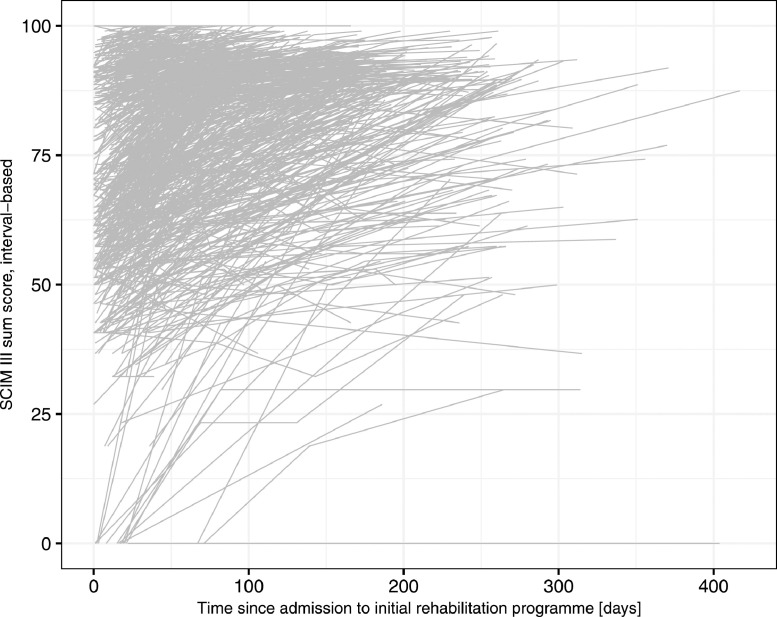
Observed individual functioning trajectories according to the interval-based SCIM III sum score.

The observed individual functioning trajectories are shown in figure 1 and separated according to their respective number of assessments during initial rehabilitation stay in supplemental figure S1 (available online only at http://www.archives-pmr.org/). The analysis of the best-fitting link function for these individual trajectories according to lowest AIC showed that the quadratic I-splines with 3 knots at percentiles performed best (supplemental fig S2, available online only at http://www.archives-pmr.org/). However, because more than half of the participants had only 2 SCIM III assessments, a sensitivity analysis excluding these participants from the sample was conducted to assess the difference between quadratic I-splines with 2 or 3 knots at percentiles (supplemental fig S3, available online only at http://www.archives-pmr.org/). Because the 95% confidence interval of the quadratic I-splines with 2 knots mostly included the change described by the function with 3 knots, the function with 2 knots was chosen to be the best-fitting link function for this sample.

**Fig 2:**
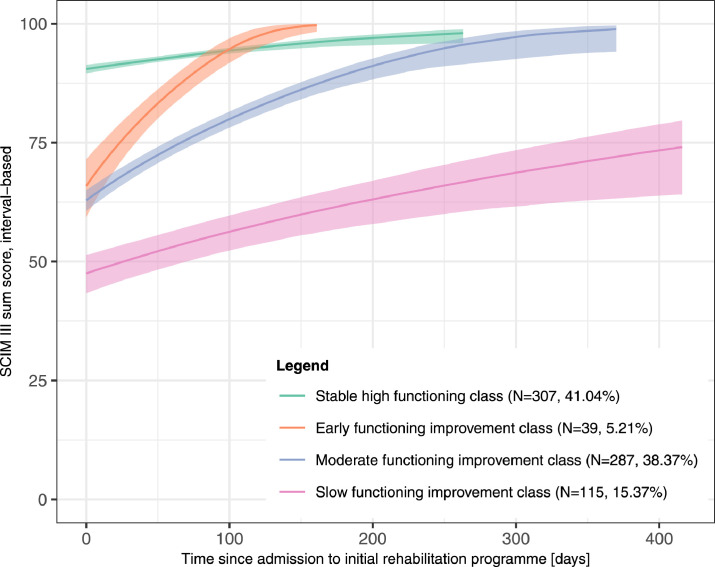
Class-specific predicted mean functioning trajectories according to the best-fitting LPMM and 95% confidence interval. Note that the class-specific predicted mean trajectories were plotted up the respective maximum observed time of assessment within each class.

**Fig 3:**
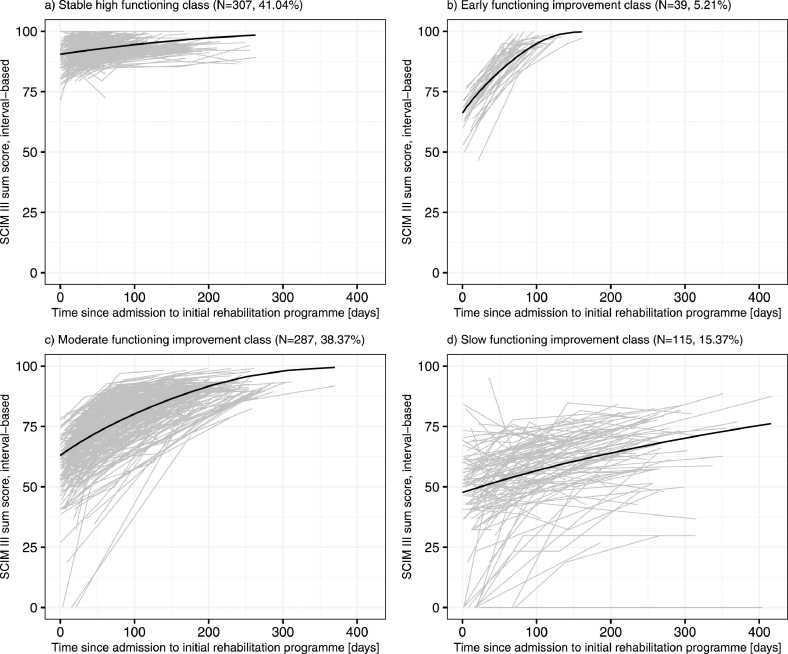
Observed individual (gray) and predicted mean (black) functioning trajectories of the best-fitting latent process mixed model for the (a) stable high functioning class (n=307; 41.04%), (b) early functioning improvement class (n=39; 5.21%), (c) moderate functioning improvement class (n=287; 38.37%), and (d) slow functioning improvement class (n=115; 15.37%).

Table 2 presents the fit characteristics of the 2 tested sets of LPMMs, and supplemental figures S4 and S5 (available online only at http://www.archives-pmr.org/) show the class-specific predicted mean trajectories identified by each model within the 2 sets, respectively. Both model sets showed overall good visual interpretability of the class-specific predicted mean functioning trajectories. Because the class-specific variability of between-person trajectories used in the second model set allows more flexibility, this set was preferred. In this set the 6-class and 5-class models included classes with <5% of the study participants and were excluded as candidates for the best-fitting LPMM. Within the remaining candidate models, BIC, SSABIC, and AIC did not clearly point to a single model. The 4-class model was preferred by SSABIC and AIC and the 3-class model by BIC. Because the addition of a fourth class to the 3-class model splits an existing class into 2 different unique classes, both of which seemed meaningful and showed satisfying sample sizes, we considered the 4-class model as best-fitting. Moreover, it showed a good entropy value of 0.80. The identified class-specific predicted mean functioning trajectories are shown in figure 2 and describe *stable high functioning* (n=307; 41.04%), *early functioning improvement* (n=39; 5.21%), *moderate functioning improvement* (n=287; 38.37%), and *slow functioning improvement* (n=115; 15.37%), respectively. Figure 3 complements the class-specific predicted mean trajectories with the respective observed individual functioning trajectories. Corresponding posteriori classification accuracy is presented in table 3. Accordingly, the LPMM shows most difficulties classifying members of the *early improvement class* with mean posterior misclassification probability of 27.36% for the *moderate improvement class*. Class-specific sample characteristics are shown in table 4, and the model parameter estimates can be found in supplemental table S1 (available online only at http://www.archives-pmr.org/).

**Table 2:** Fit characteristics of the LPMMs

Model Specifications	No. of Classes	Random Starts (Departures/Iterations)	No. of Model Parameters	BIC	SSABIC	AIC	Entropy	Class 1 Sample Size, n (%)	Class 2 Sample Size, n (%)	Class 3 Sample Size, n (%)	Class 4 Sample Size, n (%)	Class 5 Sample Size, n (%)	Class 6 Sample Size, n (%)
Set 1: LPMMs with class-invariant variability of between-person trajectories	1	100/50	8	15146.91	15121.50	15109.97	1.00	748 (100.00)					
	2	100/50	11	15027.35	14992.42	14976.56	0.83	323 (43.18)	425 (56.82)				
	3	100/50	14	14983.97	14939.52	14919.33	0.79	304 (40.64)	320 (42.78)	124 (16.58)			
	4	100/50	17	14967.75	14913.77	14889.25	0.81	269 (35.96)	45 (6.02)	117 (15.64)	317 (42.38)		
	5	100/50	20	14980.99	14917.48	14888.64	0.75	233 (31.15)	314 (41.98)	66 (8.82)	42 (5.61)	93 (12.43)	
	6*	100/50	23	15000.13	14927.10	14893.93	0.75	228 (30.48)	314 (41.98)	2 (0.27)	70 (9.36)	41 (5.48)	93 (12.43)
Set 2: LPMMs with class-specific variability of between-person trajectories	1	100/50	8	15146.91	15121.50	15109.97	1.00	748 (100.00)					
	2	100/50	12	15103.06	15064.95	15047.65	0.85	110 (14.71)	638 (85.29)				
	3	100/50	16	14979.32	14928.52	14905.45	0.77	340 (45.45)	112 (14.97)	296 (39.57)			
	4†	100/50	20	14980.76	14917.25	14888.41	0.80	39 (5.21)	115 (15.37)	307 (41.04)	287 (38.37)		
	5	100/50	24	15002.72	14926.51	14891.90	0.79	255 (34.09)	43 (5.75)	311 (41.58)	103 (13.77)	36 (4.81)	
	6	100/50	28	15025.92	14937.01	14896.63	0.77	271 (36.23)	7 (0.94)	308 (41.18)	18 (2.41)	102 (13.64)	42 (5.61)

⁎ Indicates convergence problems.

† Best-fitting LPMM.

**Table 3:** Posterior classification table of the best-fitting LPMM

Classes	n (%)	Mean Posterior Class Membership Probabilities, %			
		Stable High Functioning	Early Functioning Improvement	Moderate Functioning Improvement	Slow Functioning Improvement
Stable high functioning	307 (41.04)	93.20	1.50	5.24	0.06
Early functioning improvement	39 (5.21)	5.96	66.64	27.36	0.04
Moderate functioning improvement	287 (38.37)	3.08	3.50	87.71	5.71
Slow functioning improvement	115 (15.37)	0.20	0.01	14.49	85.31

**Table 4:** Characteristics of classes of functioning trajectories according to the best-fitting LPMM

Characteristics	Stable High Class (n=307)	Early Improvement Class (n=39)	Moderate Improvement Class (n=287)	Slow Improvement Class (n=115)
Female, n (%)	111 (36.16)	15 (38.46)	82 (28.57)	35 (30.43)
Age at SCI diagnosis, mean ± SD, y	51.77**±**17.26	54.15**±**16.84	55.09**±**19.06	61.50**±**18.37
Length of stay, mean ± SD, d	91.91**±**53.06	100.33**±**23.10	170.89**±**66.08	216.16**±**81.18
Interval-based SCIM III sum score at T1,				
median [Q_1_, Q_3_]	90.18 [86.11, 93.75]	68.78 [62.90, 74.93]	63.79 [54.50, 70.37]	49.93 [40.71, 57.37]
Missing, n (%)	18 (5.86)	1 (2.56)	10 (3.48)	11 (9.57)
Interval-based SCIM III sum score at T2,				
median [Q_1_, Q_3_]	92.53 [90.94, 94.81]	91.98 [89.98, 94.59]	84.29 [73.31, 88.33]	55.95 [49.93, 64.98]
Missing, n (%)	192 (62.54)	17 (43.59)	119 (41.46)	54 (46.96)
Interval-based SCIM III sum score at T3,				
median [Q_1_, Q_3_]	92.76 [90.92, 94.59]	94.81 [94.81, 94.81]	88.86 [83.58, 91.87]	62.60 [54.50, 69.35]
Missing, n (%)	280 (91.21)	38 (97.44)	191 (66.55)	61 (53.04)
Interval-based SCIM III sum score at T4,				
median [Q_1_, Q_3_]	95.80 [93.45, 97.75]	95.01 [93.45, 96.99]	89.65 [85.28, 91.87]	66.10 [54.50, 73.31]
Missing, n (%)	6 (1.95)	2 (5.13)	0 (0.00)	2 (1.74)
Assessment time point SCIM III T1 since admission to initial rehabilitation program,				
median [Q_1_, Q_3_], d	13.00 [4.00, 20.00]	10.50 [3.00, 16.25]	12.00 [2.00, 20.00]	9.00 [2.00, 18.25]
Missing, n (%)	18 (5.86)	1 (2.56)	10 (3.48)	11 (9.57)
Assessment time point SCIM III T2 since admission to initial rehabilitation program,				
median [Q_1_, Q_3_], d	67.00 [55.50, 75.00]	70.00 [56.00, 75.00]	69.00 [58.75, 77.00]	69.00 [60.00, 77.00]
Missing, n (%)	192 (62.54)	17 (43.59)	119 (41.46)	54 (46.96)
Assessment time point SCIM III T3 since admission to initial rehabilitation program,				
median [Q_1_, Q_3_], d	152.00 [138.50, 162.50]	141.00 [141.00, 141.00]	148.00 [137.00, 159.25]	146.00 [137.25, 158.50]
Missing, n (%)	280 (91.21)	38 (97.44)	191 (66.55)	61 (53.04)
Assessment time point SCIM III T4 since admission to initial rehabilitation program,				
median [Q_1_, Q_3_], d	75.00 [47.00, 118.00]	93.00 [77.00, 108.00]	170.00 [128.50, 201.00]	225.00 [162.00, 263.00]
Missing, n (%)	6 (1.95)	2 (5.13)	0 (0.00)	2 (1.74)
Traumatic etiology, n (%)	143 (46.58)	27 (69.23)	168 (58.54)	77 (66.96)
Level of injury at T1, n (%)				
Tetraplegia	80 (26.06)	11 (28.21)	83 (28.92)	63 (54.78)
Paraplegia	182 (59.28)	22 (56.41)	177 (61.67)	39 (33.91)
Intact	5 (1.63)	1 (2.56)	0 (0.00)	0 (0.00)
Missing	40 (13.03)	5 (12.82)	27 (9.41)	13 (11.30)
Severity of injury at T1, n (%)				
Complete	15 (4.89)	1 (2.56)	90 (31.36)	41 (35.65)
Incomplete	244 (79.48)	32 (82.05)	167 (58.19)	62 (53.91)
Normal	4 (1.30)	1 (2.56)	0 (0.00)	0 (0.00)
Missing	44 (14.33)	5 (12.82)	30 (10.45)	12 (10.43)
Associated injuries, n (%)				
No	94 (30.62)	12 (30.77)	48 (16.72)	34 (29.57)
Yes	86 (28.01)	19 (48.72)	141 (49.13)	52 (45.22)
Missing	127 (41.37)	8 (20.51)	98 (34.15)	29 (25.22)
Comorbidities before SCI diagnosis, n (%)				
No	50 (16.29)	6 (15.38)	57 (19.86)	14 (12.17)
Yes	247 (80.46)	30 (76.92)	217 (75.61)	98 (85.22)
Missing	10 (3.26)	3 (7.69)	13 (4.53)	3 (2.61)
Language of correspondence, n (%)				
German	233 (75.90)	30 (76.92)	207 (72.13)	81 (70.43)
French	63 (20.52)	9 (23.08)	71 (24.74)	22 (19.13)
Italian	6 (1.95)	0 (0.00)	6 (2.09)	8 (6.96)
Other	4 (1.30)	0 (0.00)	2 (0.70)	0 (0.00)
Missing	1 (0.33)	0 (0.00)	1 (0.35)	4 (3.48)
Insurance type, n (%)				
Health	209 (68.08)	24 (61.54)	155 (54.01)	64 (55.65)
Disability	3 (0.98)	0 (0.00)	2 (0.70)	2 (1.74)
Accident	91 (29.64)	15 (38.46)	129 (44.95)	46 (40.00)
Missing	4 (1.30)	0 (0.00)	1 (0.35)	3 (2.61)
Ward type, n (%)				
Basic	180 (58.63)	28 (71.79)	142 (49.48)	54 (46.96)
Semiprivate	76 (24.76)	2 (5.13)	86 (29.97)	39 (33.91)
Private	34 (11.07)	7 (17.95)	42 (14.63)	20 (17.39)
Missing	17 (5.54)	2 (5.13)	17 (5.92)	2 (1.74)
Partner at time of SCI diagnosis, n (%)				
No	56 (18.24)	4 (10.26)	43 (14.98)	20 (17.39)
Yes	148 (48.21)	21 (53.85)	108 (37.63)	28 (24.35)
Missing	103 (33.55)	14 (35.90)	136 (47.39)	67 (58.26)
Cardiovascular conditions and complications at T1 since SCI diagnosis, n (%)				
No	233 (75.90)	25 (64.10)	198 (68.99)	68 (59.13)
Yes	73 (23.78)	14 (35.90)	87 (30.31)	47 (40.87)
Missing	1 (0.33)	0 (0.00)	2 (0.70)	0 (0.00)
Pulmonary conditions and complications at T1 since SCI diagnosis, n (%)				
No	257 (83.71)	28 (71.79)	185 (64.46)	49 (42.61)
Yes	45 (14.66)	11 (28.21)	101 (35.19)	65 (56.52)
Missing	5 (1.63)	0 (0.00)	1 (0.35)	1 (0.87)
Ventilation assistance at T1 since SCI diagnosis, n (%)				
No	298 (97.07)	37 (94.87)	258 (89.90)	81 (70.43)
Yes	5 (1.63)	2 (5.13)	27 (9.41)	34 (29.57)
Missing	4 (1.30)	0 (0.00)	2 (0.70)	0 (0.00)
Normal defecation at T1 since SCI diagnosis, n (%)				
No	82 (26.71)	15 (38.46)	149 (51.92)	47 (40.87)
Yes	131 (42.67)	8 (20.51)	15 (5.23)	5 (4.35)
Missing	94 (30.62)	16 (41.03)	123 (42.86)	63 (54.78)
Urinary tract infection at T1 since SCI diagnosis, n (%)				
No	177 (57.65)	16 (41.03)	103 (35.89)	38 (33.04)
Yes	47 (15.31)	11 (28.21)	69 (24.04)	18 (15.65)
Missing	83 (27.04)	12 (30.77)	115 (40.07)	59 (51.30)
Pressure injury at T1 since SCI diagnosis, n (%)				
No	208 (67.75)	23 (58.97)	125 (43.55)	36 (31.30)
Yes	13 (4.23)	3 (7.69)	55 (19.16)	22 (19.13)
Missing	86 (28.01)	13 (33.33)	107 (37.28)	57 (49.57)
Pain at T1 in the past week, n (%)				
No	48 (15.64)	7 (17.95)	28 (9.76)	7 (6.09)
Yes	127 (41.37)	15 (38.46)	97 (33.80)	21 (18.26)
Missing	132 (43.00)	17 (43.59)	162 (56.45)	87 (75.65)

Abbreviations: Q_1_, 1st quartile; Q_3_, 3rd quartile; T1-T4, time point of assessment within the SwiSCI Inception Cohort Study.

### Predictors of class membership

Table 5 presents the results of the multinomial logistic regression analysis (n=709; AIC, 1401.83) with the *slow functioning improvement class* used as a reference class. Thereby, the coefficients of the regression analysis describe the estimated change of the relative logit of being in a specific class compared with the reference class. The coefficients are to be interpreted for 1 unit change in a continuous predictor variable and for changing from the reference category to a specific other category in a categorical predictor variable, holding all other respective predictor variables constant. Accordingly, the likelihood of being in any other than the reference class is decreased by higher age and the occurrence of pulmonary conditions and complications, whereas a lower injury level of or an incomplete injury increased this likelihood. Moreover, ventilation assistance decreased the likelihood of being in the *stable high* or the *moderate improvement class* compared with the reference class, and having a private ward type decreased the likelihood of being in the *early improvement class* compared with the reference class. Of the remaining predictors, sex, language of correspondence, etiology, comorbidities before SCI, cardiovascular conditions and complications, and insurance type did not show any significant associations.

**Table 5:** Multinomial logistic regression of class membership for best-fitting LPMM (n=709)

Variable	Estimates (95% confidence interval)		
	Stable High Functioning Class (Ref = Slow Functioning Improvement Class)	Early Functioning Improvement Class (Ref = Slow Functioning Improvement Class)	Moderate Functioning Improvement Class (Ref = Slow Functioning Improvement Class)
Intercept	2.15* (0.51-3.78)	–2.64 (–5.65 to 0.37)	2.24† (0.80-3.67)
Age	–0.06‡ (–0.08 to –0.04)	–0.04† (–0.07 to –0.01)	–0.02* (–0.04 to –0.00)
Female (Ref = Male)	–0.48 (–1.08 to 0.12)	–0.13 (–1.01 to 0.74)	–0.47 (–1.02 to 0.09)
Language of correspondence, French (Ref = German)§	–0.64 (–1.34 to 0.07)	–0.56 (–1.58 to 0.46)	–0.07 (–0.70 to 0.56)
Traumatic etiology (Ref = Nontraumatic)	–0.43 (–1.16 to 0.30)	0.86 (–0.19 to 1.92)	–0.63 (–1.30 to 0.04)
Level of injury, paraplegia (Ref = Tetraplegia)‖	1.79‡ (1.18-2.40)	2.03‡ (1.13-2.94)	1.26‡ (0.71-1.81)
Severity of injury, incomplete (Ref = Complete)¶	3.56‡ (2.74-4.39)	4.34‡ (2.25-6.43)	0.87† (0.28-1.46)
Comorbidities before SCI, yes (Ref = No)	–0.18 (–1.04 to 0.68)	–0.04 (–1.29 to 1.20)	–0.43 (–1.19 to 0.34)
Cardiovascular conditions and complications, yes (Ref = No)	0.04 (–0.59 to 0.67)	0.61 (–0.34 to 1.56)	0.07 (–0.49 to 0.63)
Pulmonary conditions and complications, yes (Ref = No)	–1.50‡ (–2.13 to –0.87)	–1.12* (–2.05 to –0.18)	–0.63* (–1.18 to –0.08)
Insurance type, accident (Ref = Health)#	–0.57 (–1.40 to 0.26)	–0.25 (–1.41 to 0.90)	0.33 (–0.41 to 1.06)
Ward type, private (Ref = Basic)⁎⁎	–0.53 (–1.12 to 0.06)	–1.46† (–2.44 to –0.49)	–0.24 (–0.77 to 0.28)
Ventilation assistance, yes (Ref = No)	–2.20‡ (–3.31 to –1.08)	–1.26 (–2.91 to 0.40)	–0.83* (–1.51 to –0.15)

⁎ *P*<.05.

† *P*<.01.

‡ *P*<.001.

§ Participants with observations in the response categories “Italian” or “other” were excluded from the analysis.

‖ Participants with observations in the response category “intact” were excluded from the analysis.

¶ Participants with observations in the response category “normal” were excluded from the analysis.

# Participants with observations in the response categories “disability” or “self-pay” were excluded from the analysis.

⁎⁎ Response categories “semiprivate” and “private” were collapsed.

The results of the corresponding sensitivity analysis (n=546; AIC, 1100.19) are shown in supplemental table S2 (available online only at http://www.archives-pmr.org/). Compared with the results in table 5, supplemental table S2 (available online only at http://www.archives-pmr.org/) also shows a decreased likelihood of being in the *stable high class* for private ward type and of being in the *moderate improvement class* for having a traumatic etiology*,* compared with the reference class. The occurrence of pulmonary conditions and complications was only associated with a lowered likelihood for being in the *stable high class.* Overall, age, injury level, injury severity, and ventilator assistance appeared to be robust predictors of class membership across both analyses within the present sample.

## Discussion

This study revealed 4 distinct classes of functioning trajectories in individuals with SCI who underwent initial rehabilitation in specialized centers in Switzerland. According to the class-specific predicted mean functioning trajectories, the identified classes describe *stable high functioning* (n=307; 41.04%), *early functioning improvement* (n=39; 5.21%), *moderate functioning improvement* (n=287; 38.37%), and *slow functioning improvement* (n=115; 15.37%), respectively. To our knowledge, this is the first study identifying classes of functioning trajectories in individuals with SCI according to SCIM III sum scores. Given the limited body of empirical knowledge on this topic, there is a limited extent to which our results can be compared with other studies, such as individual growth curve models of change in functioning outcomes according to the FIM^30^, ^31^, ^32^ or the identification of classes of trajectories of different outcomes in SCI.^12^, ^13^, ^14^, ^15^, ^16^, ^17^, ^18^

Although LPMMs and individual growth curve models share some commonalities, the latter do not incorporate any assumption about underlying, unobserved classes. Thus, individual growth curve models can be used to study individual change in functioning, whereas LPMMs can be used to study classes of similar change in functioning. Pretz et al^32^ have described several potential applications of individual growth curve models in practice such as rehabilitation goal setting, intervention planning, and individual patient benchmarking. Nevertheless, we believe that for the monitoring of functioning throughout the rehabilitation process, it is also meaningful to be able to see how an individual patient is changing in comparison to similar patients. The identification of classes of functioning trajectories can be a first step toward enabling such monitoring. However, further research is needed for it to be implemented in practice.

Trajectory studies on outcomes such as life satisfaction^15^ or employment status^14^ have shown that independence in performing ADL assessed by the FIM is a predictor of the respective classes of trajectories. Although we do not know if these findings also hold for the SCIM, we believe that the importance of the longitudinal relationships between functioning and other outcomes should be investigated in future research.

Multinomial logistic regression showed that age, injury level, injury severity, and ventilator assistance are robust predictors that can distinguish between the identified classes of functioning trajectories in the present sample. Given the exploratory nature of our study, these findings are preliminary and need to be confirmed by other studies. Although age, injury level, and severity have also been identified as relevant predictors of the SCIM in previous studies,^10^^,^^33^^,^^34^ these findings are only comparable to a limited extent because a relevant predictor for SCIM outcomes at a specific endpoint is not necessarily relevant for classes of change according to SCIM within a specific time frame. Nevertheless, having a look beyond the predictors assessed within our study, variables such as smoking status, different strength values, acute care length of stay, postacute length of stay, occurrence of complications, and SCIM score at administration have been significantly associated with SCIM III outcomes up to 1 year after SCI in respective other studies^10^^,^^33^^,^^35^ and thus should be checked as predictors of class membership of functioning trajectories in future research.

From a statistical point of view, it is important to validate and, if possible, update the identified classes of functioning trajectories in larger study populations and study designs that include more assessment time points, outcome measures, and predictors. Moreover, the number of classes might increase with increasing sample sizes. This is reflected in figure 3, which reveals that the *slow functioning improvement class* covers a wide range of observed individual functioning trajectories. This class might be split into new distinct classes for an increased sample size including more observations on low individual functioning trajectories. From a practical point of view, it is essential to evaluate with qualitative studies the meaning and value of the identified classes of functioning trajectories for clinical practice from the perspective of rehabilitation professionals. In the future, the findings of the present study might assist in developing clinical prediction models of functioning able to assign newly injured individuals to a specific class of functioning trajectories. In addition, classes of functioning trajectories, analyzed together with information on rehabilitation practices may support monitoring patient outcomes, contribute to the development of patient pathways for SCI initial rehabilitation, and support rehabilitation planning and management.

### Study limitations

The limitations of this study are consistent with the use of existing data for secondary analysis in which no influence is possible on the initial data collection. First, there are limitations related to the SwiSCI and the corresponding operationalization of functioning used in the present study. SCIM is an instrument representing independence in ADL and does not include restrictions in “activities and participation” as defined in the International Classification of Functioning, Disability and Health. Despite the fact that it was specifically developed for individuals with SCI and has been demonstrated to be superior to the FIM, there are some disadvantages to mention within the scope of this study, including its proneness for floor and ceiling effects. In addition, although the SwiSCI Inception Cohort includes a comprehensive study design, the number of available SCIM III assessments and potential predictor variables in rehabilitation stay restricted the study results. For example, the included predictor variables level of injury, pulmonary conditions and complications, and ventilator assistance cover related characteristics of individuals with SCI. Furthermore, a selection bias could have occurred owing to the exclusion of study participants with implausible time points of assessments and individuals with less than 2 SCIM III observations during initial rehabilitation stay. Country specific differences with regard to clinical rehabilitation practice (eg, availability, eligibility, comprehensiveness, and duration of inpatient rehabilitation) might further limit the generalizability of the results. Second, class membership probabilities might depend on participant characteristics, and results can change if such covariates are included within LPMMs. However, Hu et al have shown that the approach used in this study is acceptable with a large sample size and good class separation.^36^ Generally, classification accuracy of the final LPMM will improve if the available number of the *early functioning improvement class* members will increase. Third, the multinomial logistic regression of potential predictors of class membership did not take into account the classification errors of the LPMM. This leads to bias in the regression models and, in general, true effects might be underestimated.^27^ Furthermore, the small sample size of the *early functioning improvement class* resulted in small numbers of observations in response categories of some predictors within this class, such as ward types, and might influence the performed analysis and corresponding results.

## Conclusions

The present study establishes a foundation for future research on the course of functioning of individuals with SCI in initial rehabilitation by identifying classes of functioning trajectories. This supports the development of specifically tailored rehabilitation programs and prediction models, which can be integrated into clinical rehabilitation planning.

## Suppliers

a. R, version 3.6.0; R Foundation for Statistical Computing.
b. lcmm R package, version 1.8.1; Proust-Lima C, Philipps V, Liquet B.
c. missForest R package, version 1.4; Stekhoven DJ, Bühlmann P.
d. nnet R package, version 7.3-12; Venables WN, Ripley BD.

## Appendix Supplementary materials

Image, application 1

Image, application 2

Image, application 3

Image, application 4

Image, application 5

Image, application 6

Image, application 7

Image, application 8
