# Supplementary material for: Identification of Classes of Functioning Trajectories and Their Predictors in Individuals With Spinal Cord Injury Attending Initial Rehabilitation in Switzerland
Source: Arch Rehabil Res Clin Transl. 2021 Mar 15;3(2):100121. doi: 10.1016/j.arrct.2021.100121 (PMC8212008; doi:10.1016/j.arrct.2021.100121)
Supplement: Supplementary file 1 [file mmc1.pdf]

**Supplemental Appendix S1**

Within this technical appendix, we describe the latent process mixed model (LPMM) analysis procedure in full detail and present the R syntax of the final LPMM.

**A) LPMM analysis procedure**

Following Proust-Lima et al.[1] the analysis included two steps:

(1) A set of three LPMMs with different parameterized link functions – linear function and quadratic I-splines functions with two or three knots at percentiles - were fitted to identify the best-fitting link function able to account for non-normal and bounded longitudinal outcomes.[1]

*Model specification information:* All models followed equal specifications with respect to the number of latent classes (one), the effect for time of assessment (linear random effects including random intercept and slope) and correlated differences in the change of the outcome (unstructured random effects variance-covariance matrix).

(2) Two sets of six LPMMs, each with an increasing number of latent classes (one to six), were fitted to identify the number of classes of functioning trajectories.

*Model specification information*: The difference between the set of models was the specification of the random effects variance-covariance matrix, which was set to be class-invariant (fixed across classes) in the first, and class-specific (proportionally varying across classes) in the second set of models. The common settings of both model sets included linear random effects for time of assessment, unstructured random effects variance-covariance matrix, class-specific patterns of change on the outcome (linear mixture effects for time of assessment), and the best-fitting link function from step one.

In both steps, all fitted models correspond to unconditional models, i.e. no covariates were integrated.

**B) R syntax of the final LPMM**

The used R syntax for the estimation of the final LPMM was based on the R Package *lcmm version 1.8.1*[1] and looks as follows:

```
m4spl2q    <-   gridsearch(rep = 100, maxiter = 50, minit = m1spl2q,
                lcmm(SCIM_rasch~days_SCIM_adm, random=~days_SCIM_adm, mixture=~days_SCIM_adm,
                subject='id_swisci_num', ng=4, nwg = T, idiag = F, data=data_long, link='2-quant-splines'))
```

*m4spl2q* R object where results of the final model estimation are stored

*gridsearch(..)* R function to perform a grid of random initial values for model estimation. Used options are:

- *rep* Number of random starting values departures, which is set to *100*.
- *maxiter* Number of iterations, which is set to *50*.
- *minit* Random starting values, which is generated based on a previously estimated model (*m1spl2q*) corresponding to *m4spl2q* with one class of functioning trajectories only.

*lcmm(..)* R function to estimate the final LPMM model. Used options are:

- *SCIM_rasch~days_SCIM_adm* Fixed effects within the estimated model, which are set to linear fixed effects for the time of assessment variable (*days_SCIM_adm*) on the repeated functioning measures (*SCIM_rasch*).
- *random* Random effects within the estimated model, which are set to linear random effects including random intercept and slope for the time of assessment variable (*days_SCIM_adm*).
- *mixture* Mixture effects within the estimated model, which are set to linear mixture effects for the time of assessment variable (*days_SCIM_adm*).
- *subject* Participant identification number, which is set to the respective variable within the prepared SwiSCI Inception Cohort data set (*id_swisci_num*).
- *ng* Number of classes of functioning trajectories used for model estimations, which is set to *4* for the final model.
- *nwg* Setting for the random effects variance-covariance matrix, which was specified to be class-specific (TRUE), i.e. allowing the between-participants variability in trajectories to be different across classes.
- *idiag* Setting for the random effects variance-covariance matrix, which was specified to be unstructured (*FALSE*), i.e. allowing correlated random effects.
- *data* Data set used for model estimations (needs to be in long format), which is set to the prepared SwiSCI Inception Cohort data set (*data_long*).
- *link* Parametrized link function used for model estimation, which is set to the quadratic I-splines functions with two knots at percentiles (*2-quant-splines*).

---

[1]Proust-Lima C, Philipps V, Liquet B. Estimation of Extended Mixed Models Using Latent Classes and Latent Processes: The R Package lcmm. Journal of Statistical Software 2017;78(2):1-56.
