# Supplementary material for: Identification of Classes of Functioning Trajectories and Their Predictors in Individuals With Spinal Cord Injury Attending Initial Rehabilitation in Switzerland
Source: Arch Rehabil Res Clin Transl. 2021 Mar 15;3(2):100121. doi: 10.1016/j.arrct.2021.100121 (PMC8212008; doi:10.1016/j.arrct.2021.100121)

Supplemental Figure S1

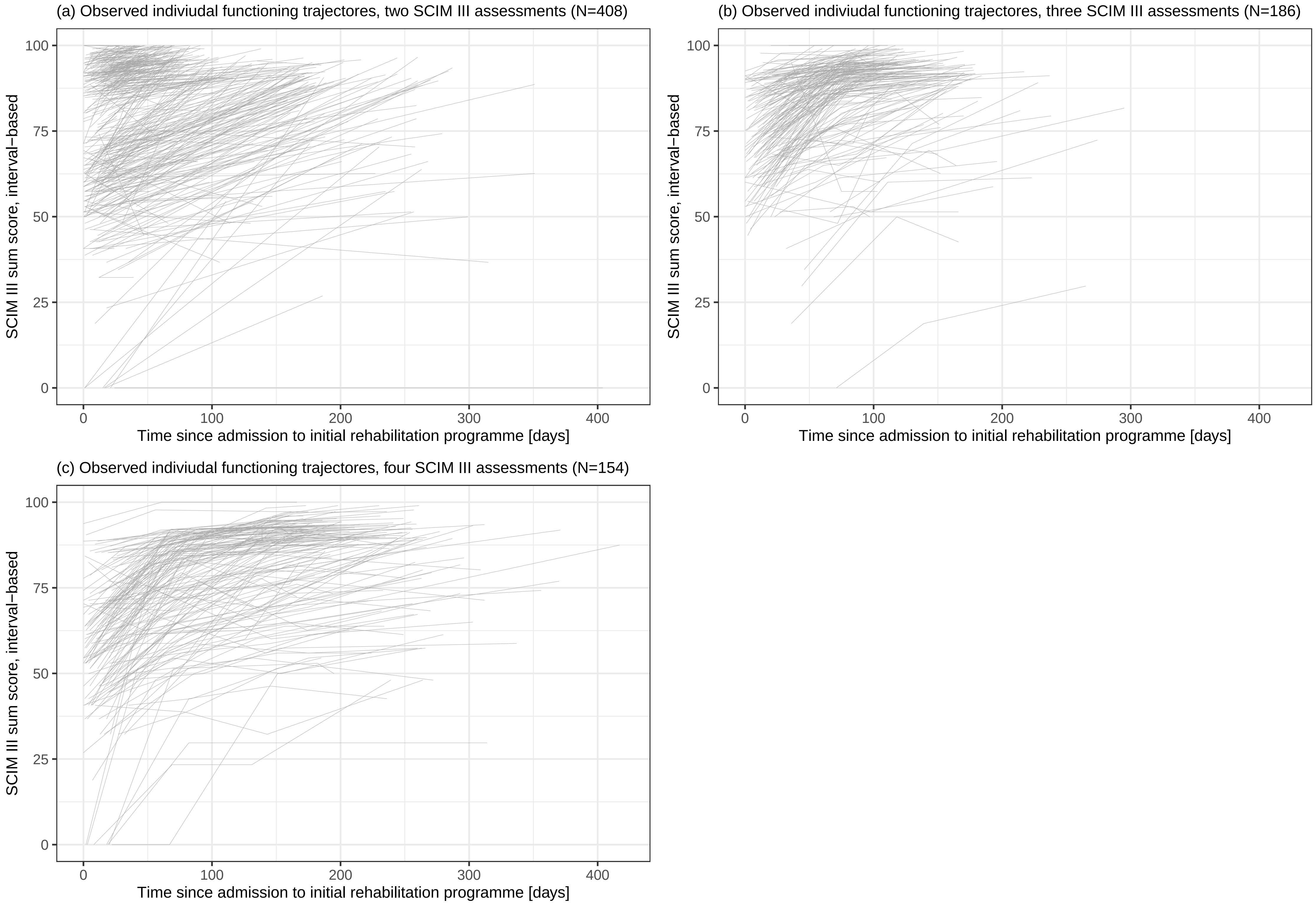

Supplement: Supplementary file 2 [file mmc2.pdf]
