# Supplementary figures and images for: Identification of Classes of Functioning Trajectories and Their Predictors in Individuals With Spinal Cord Injury Attending Initial Rehabilitation in Switzerland
Source: Arch Rehabil Res Clin Transl. 2021 Mar 15;3(2):100121. doi: 10.1016/j.arrct.2021.100121 (PMC8212008; doi:10.1016/j.arrct.2021.100121)

Supplemental Figure S2 Estimated parameterized link functions

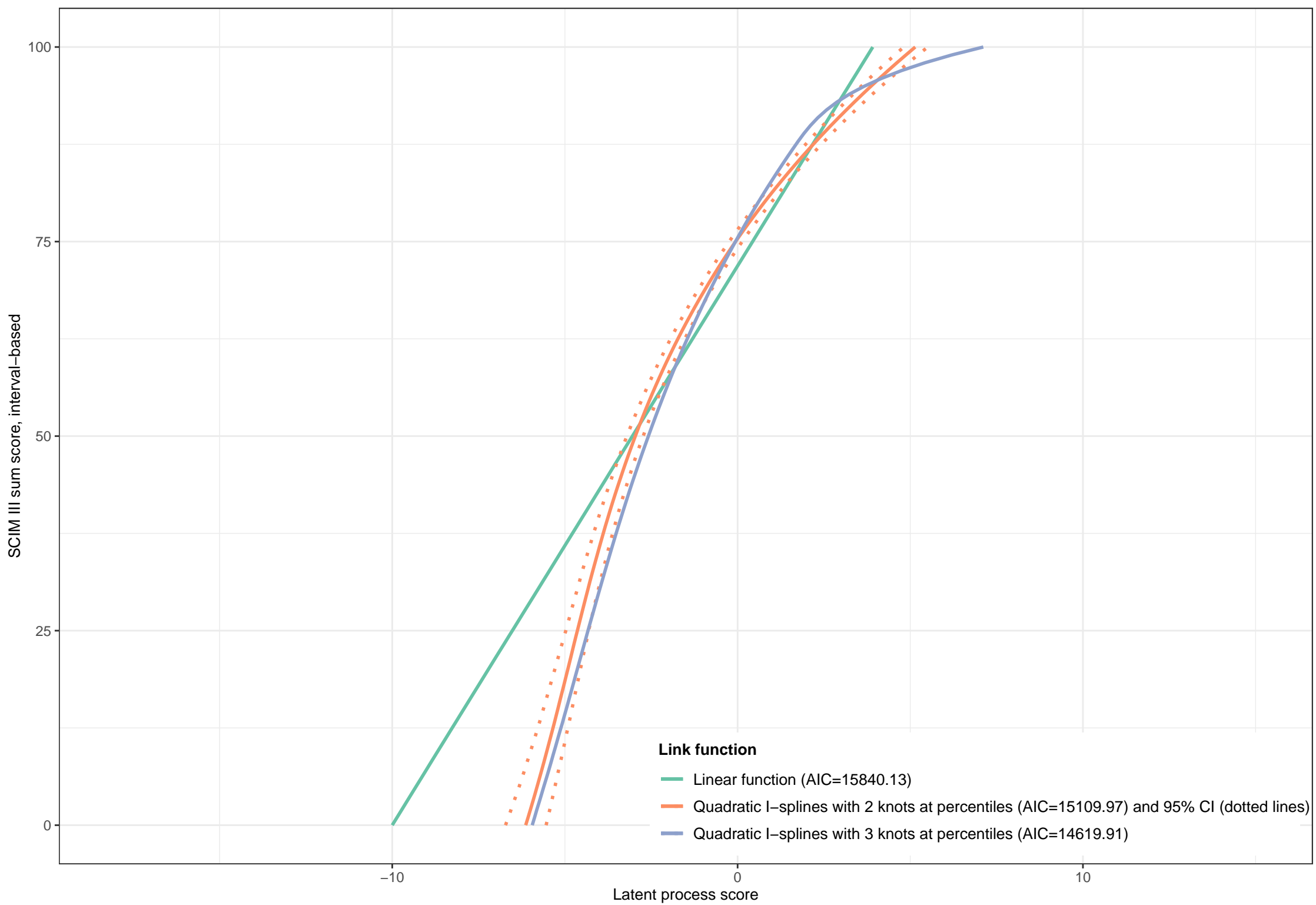

Supplement: Supplementary file 3 [file mmc3.pdf]

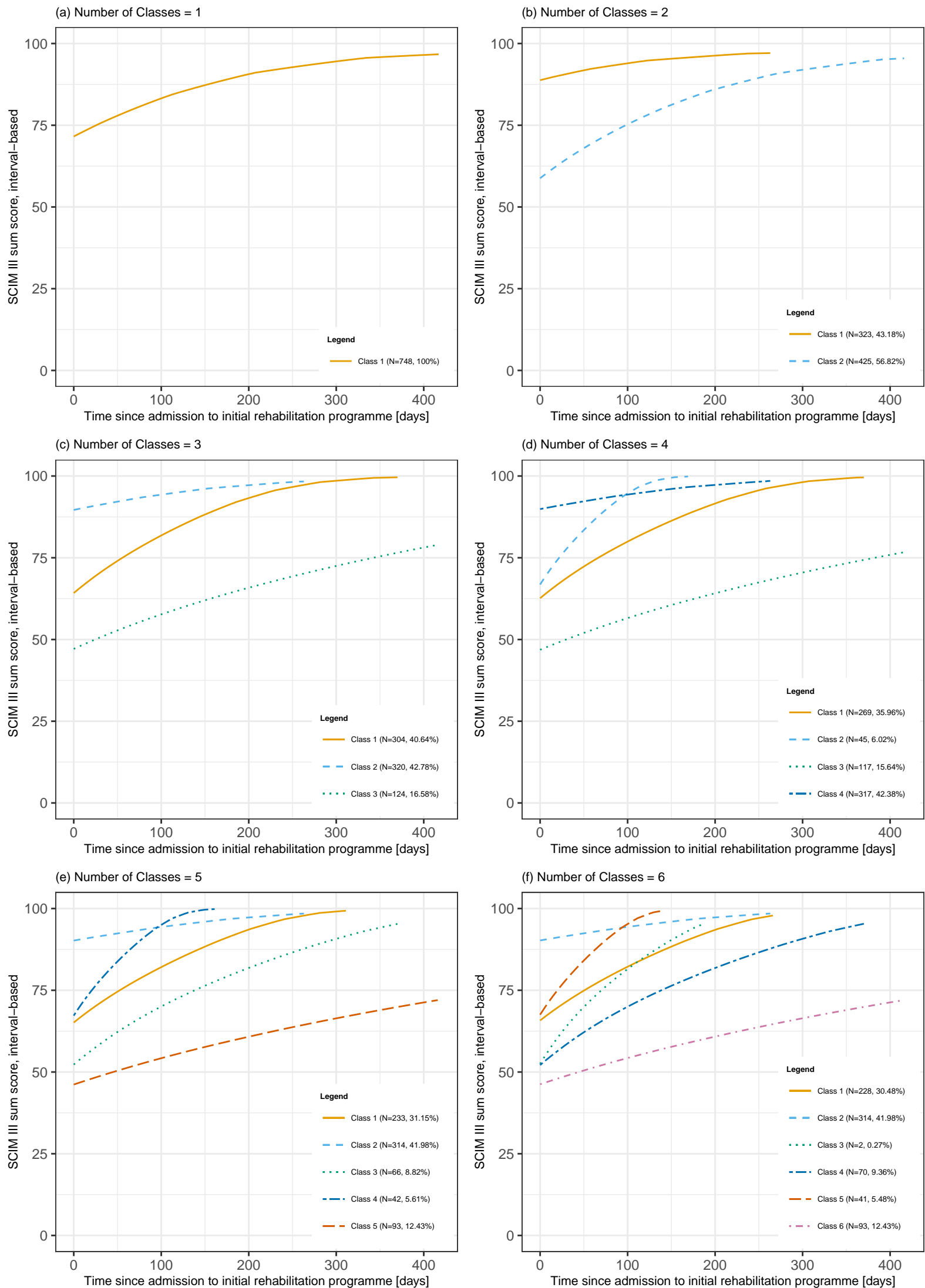

Supplement: Supplementary file 5 [file mmc5.pdf]

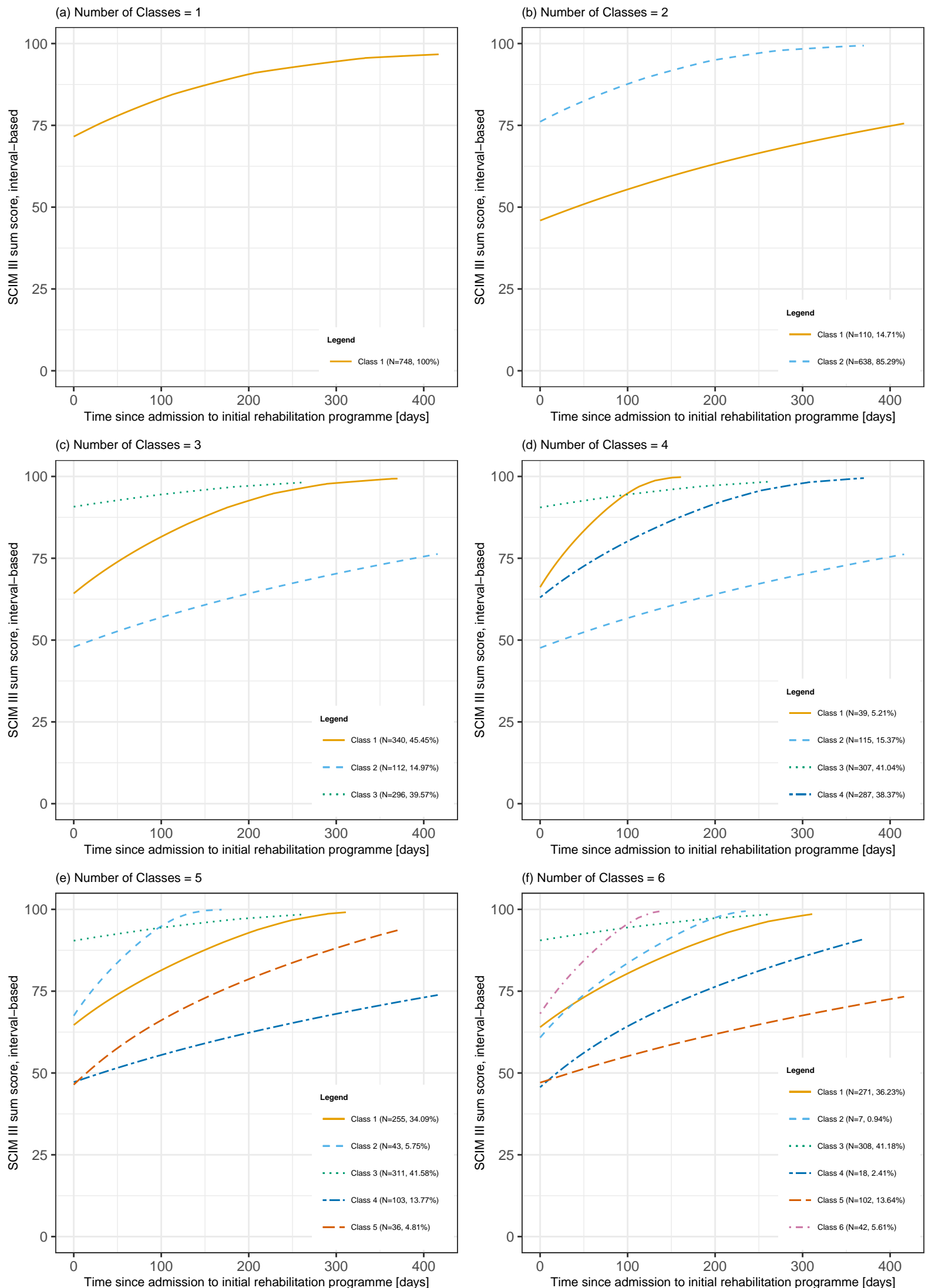

Supplement: Supplementary file 6 [file mmc6.pdf]
