# Supplementary material for: Identification of Classes of Functioning Trajectories and Their Predictors in Individuals With Spinal Cord Injury Attending Initial Rehabilitation in Switzerland
Source: Arch Rehabil Res Clin Transl. 2021 Mar 15;3(2):100121. doi: 10.1016/j.arrct.2021.100121 (PMC8212008; doi:10.1016/j.arrct.2021.100121)

Supplemental Figure S3 Estimated parameterized link functions (sensitivity analysis based on participants with three or four SCIM III assessments)

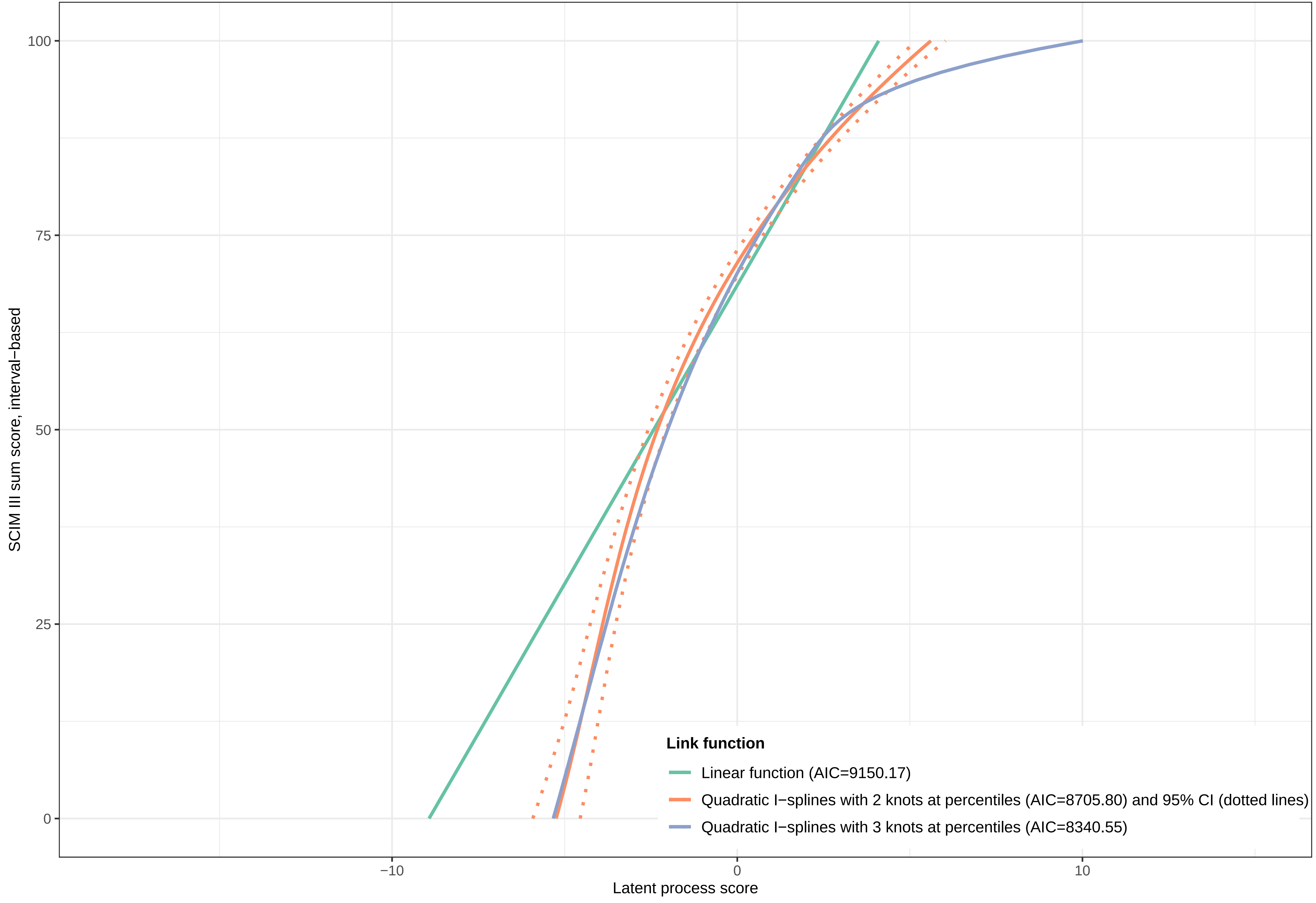

Supplement: Supplementary file 4 [file mmc4.pdf]
