# Supplementary material for: Identification of Classes of Functioning Trajectories and Their Predictors in Individuals With Spinal Cord Injury Attending Initial Rehabilitation in Switzerland
Source: Arch Rehabil Res Clin Transl. 2021 Mar 15;3(2):100121. doi: 10.1016/j.arrct.2021.100121 (PMC8212008; doi:10.1016/j.arrct.2021.100121)
Supplement: Supplementary file 7 [file mmc7.pdf]

Supplemental Table S1 Estimated parameters and posterior classification table for the best-fitting latent process mixed model

A) Fixed effects in the class-membership model (Ref=class 4)

Parameter	Coefficient	SE	P-value
Intercept class 1	-1.983	0.332	0.000
Intercept class 2	-0.945	0.178	0.000
Intercept class 3	0.008	0.111	0.943

Note. SE, standard error; class 1, early functioning improvement class; class 2, slow functioning improvement class; class 3, stable high functioning class.

B) Fixed effects of the longitudinal model

Parameter	Coefficient	SE	P-value
Intercept class 1 (not estimated)	0.000	-	-
Intercept class 2	-1.752	0.399	0.000
Intercept class 3	4.244	0.383	0.000
Intercept class 4	-0.318	0.393	0.419
Time of assessment Class 1	0.055	0.005	0.000
Time of assessment Class 2	0.008	0.001	0.000
Time of assessment Class 3	0.010	0.001	0.000
Time of assessment Class 4	0.025	0.001	0.000

Note. SE, standard error; class 1, early functioning improvement class; class 2, slow functioning improvement class; class 3, stable high functioning class; class 4, moderate functioning improvement class.

C) Variance-covariance matrix of the random effects for class 4

	Intercept	Time of assessment
Intercept	1.12249	
Time of assessment	0.00228	0.00001

Note. Class 4, moderate functioning improvement class.

D) Proportional coefficients for variance-covariance matrix of the random effects

	Coefficient	SE
Class 1	0.801	0.259
Class 2	0.933	0.147
Class 3	0.680	0.104
Residual SE (not estimated)	1	-

Note. SE, standard error; class 1, early functioning improvement class; class 2, slow functioning improvement class; class 3, stable high functioning class.

E) Parameters of the link function (quadratic I-splines with knots 0 and 100)

Parameter	Coefficient	SE	P-value
I-splines1	-4.305	0.429	0.000
I-splines2	1.188	0.102	0.000
I-splines3	-0.000	0.015	0.992
I-splines4	3.084	0.043	0.000

Note. SE, standard error.
