# Supplementary material for: Identification of Classes of Functioning Trajectories and Their Predictors in Individuals With Spinal Cord Injury Attending Initial Rehabilitation in Switzerland
Source: Arch Rehabil Res Clin Transl. 2021 Mar 15;3(2):100121. doi: 10.1016/j.arrct.2021.100121 (PMC8212008; doi:10.1016/j.arrct.2021.100121)
Supplement: Supplementary file 8 [file mmc8.pdf]

Supplemental Table S2 Sensitivity analysis of multinomial logistic regression of class membership for best-fitting latent process mixed model (N=546).

	Estimates (95% CI)		
	Stable high functioning class (Ref. = Slow functioning improvement class)	Early functioning improvement class (Ref. = Slow functioning improvement class)	Moderate functioning improvement class (Ref. = Slow functioning improvement class)
Intercept	2.47** (0.60, 4.34)	-2.27 (-5.59, 1.06)	2.31** (0.64, 3.97)
Age	-0.06*** (-0.08, -0.04)	-0.04* (-0.07, -0.01)	-0.02* (-0.05, -0.00)
Sex=Female (Ref=Male)	-0.38 (-1.05, 0.29)	0.02 (-0.98, 1.01)	-0.58 (-1.21, 0.04)
Language of correspondence=French (Ref=German)[a]	-0.59 (-1.40, 0.23)	-0.73 (-1.98, 0.52)	-0.07 (-0.81, 0.66)
Aetiology=Traumatic (Ref=Non-traumatic)	-0.67 (-1.48, 0.15)	0.74 (-0.50, 1.97)	-1.01** (-1.77, -0.25)
Level of injury=Paraplegia (Ref.=Tetraplegia)[b]	1.69*** (1.00, 2.38)	1.68** (0.67, 2.69)	1.32*** (0.69, 1.96)
Severity of injury=Incomplete (Ref.=Complete)[c]	3.28*** (2.36, 4.20)	3.77*** (1.63, 5.91)	0.93** (0.25, 1.60)
Comorbidities before SCI=Yes (Ref=No)	0.08 (-0.89, 1.05)	0.07 (-1.33, 1.47)	-0.10 (-0.98, 0.77)
Cardiovascular conditions and complications=Yes (Ref=No)	0.09 (-0.61, 0.79)	0.77 (-0.28, 1.82)	0.04 (-0.59, 0.68)
Pulmonary conditions and complications=Yes (Ref=No)	-1.13** (-1.83, -0.42)	-0.87 (-1.92, 0.18)	-0.42 (-1.04, 0.20)
Insurance type=Accident (Ref.=Health)[d]	-0.49 (-1.42, 0.44)	-0.02 (-1.33, 1.29)	0.58 (-0.24, 1.40)
Ward type=Private (Ref.=Basic)[e]	-0.69* (-1.36, -0.03)	-1.61** (-2.72, -0.51)	-0.28 (-0.88, 0.32)
Ventilation assistance=Yes (Ref=No)	-2.68*** (-4.06, -1.31)	-1.25 (-2.97, 0.46)	-0.92* (-1.69, -0.14)

NOTE. CI, confidence interval; SCI, spinal cord injury; [a]participants with observations in the response categories "Italian" or "other" were excluded from the analysis; [b]participants with observations in the response category "intact" were excluded from the analysis; [c]participants with observations in the response category "normal" were excluded from the analysis; [d]participants with observations in the response categories "disability" or "self-pay" were excluded from the analysis; [e]Response categories "semi-private" and "private" were collapsed; *p<0.05; **p<0.01; ***p<0.001.
